# The short- and long-term changes of upper airway and alar in nongrowing patients treated with Mini-Implant Assisted Rapid Palatal Expansion (MARPE): a systematic review and meta-analysis

**DOI:** 10.1186/s12903-023-03344-w

**Published:** 2023-10-29

**Authors:** Cuiyu Liu, Kaixin Wang, Chunmiao Jiang, Yang Zhao, Yiyan Zhang, Qianwen Zhang, Cunhui Fan, Yang Liu

**Affiliations:** 1 Department of Orthodontics, The Affiliated Hospital of Qingdao University, Qingdao, 266003 China; 2 School of Stomatology, Qingdao University, Qingdao, 266023 China

**Keywords:** Transverse maxillary deficiency, Mini-Implant Assisted Rapid Palatal Expansion (MARPE), Maxillary skeletal expansion, Palatal expansion technique, Upper airway volume

## Abstract

**Objective:**

This study aims to assess the short- and long-term changes in the upper airway and alar width after mini-implant -assisted rapid palatal expansion (MARPE) in nongrowing patients.

**Methods:**

Five electronic databases (PubMed, Scopus, Embase, Web of Science, and Cochrane Library) were searched up to 2 August, 2023 based on the PICOS principles. The main outcomes were classified into three groups: 1) nasal cavity changes, 2) upper airway changes and 3) alar changes. The mean difference (MD) and 95% confidence intervals (CI) were used to assess these changes. Heterogeneity tests, subgroup analyses, sensitivity analyses, and publication bias were also analyzed.

**Result:**

Overall, 22 articles were included for data analysis. Nasal cavity width (WMD: 2.05 mm; 95% CI: 1.10, 3.00) and nasal floor width (WMD: 2.13 mm; 95% CI: 1.16, 3.11) increased significantly. While palatopharyngeal volume (WMD: 0.29 cm^3^, 95% CI: -0.44, 1.01), glossopharyngeal volume (WMD: 0.30 cm^3^, 95% CI: -0.29, 0.89) and hypopharyngeal volume (WMD: -0.90 cm^3^; 95% CI: -1.86, 0.06) remained unchanged, nasal cavity volume (WMD: 1.24 cm^3^, 95% CI: 0.68, 1.81), nasopharyngeal volume (MD: 0.75 cm^3^, 95% CI: 0.44, 1.06), oropharyngeal volume (WMD: 0.61 cm^3^, 95% CI: 0.35, 0.87), and total volume of the upper airway (WMD: 1.67 cm^3^, 95% CI: 0.68, 2.66) increased significantly. Alar width (WMD: 1.47 mm; 95% CI: 0.40, 2.55) and alar base width (WMD: 1.54 mm; 95% CI: 1.21, 1.87) also increased.

**Conclusion:**

MARPE can increase nasal cavity width, nasal cavity volume, nasopharyngeal volume and oropharyngeal volume for nongrowing patients, but has no significant effect on hypopharyngeal volume. In addition, the alar width also increased. However, the studies included in this meta-analysis were mainly retrospective, nonrandomized and small in number, so the findings should be interpreted with caution and high-quality RCTs need to be studied.

**Supplementary Information:**

The online version contains supplementary material available at 10.1186/s12903-023-03344-w.

## Background

Maxillary transverse deficiency (MTD) is a kind of congenital or acquired developmental disorder, that has been reported to affect 8.0%-23.3% of patients in children and teenagers and 9.4% in adults [1]. It clinically manifests as unilateral and bilateral posterior crossbite, arch narrowing, dentition crowding, etc. Studies have reported that MTD can also cause upper airway narrowing, facial soft tissue irregularities, chewing dysfunction, pronunciation disorder, sagittal maxillary hypoplasia, and obstructive sleep apnoea-hypopnea syndrome (OSAHs) [2, 3].

Rapid palatal expansion (RPE) can produce skeletal and dental effects and alleviate the deficiency of lateral development for on-growing patients whose midpalate suture has not yet fused, especially for children and adolescents under 15 years of age [4, 5]. However, for nongrowing patients whose midpalate suture has been fused, potential limitations and side effects of conventional RME have been reported, such as expansion failure or limited skeletal expansion, instability of results, pain, tissue swelling, buccal crown tipping, gingival recession, buccal root resorption, and ulceration [6, 7].

Surgically assisted RPE (SARPE) has been used to overcome the abovementioned limitations [8]. However, SARPE is an invasive procedure that is complex, swollen, and painful. Uncomfortable in the surgical area and high costs make it difficult for most patients to accept [9, 10]. In recent years, mini-implant assisted rapid palatal expansion (MARPE) has been favored by orthodontists and patients because of its small trauma and low cost [7]. It is mainly divided into 2 parts, 1) 2 or 4 mini-implants are distributed on both sides of the midpalate suture, which can penetrate the unilateral or bilateral bone cortex or nasal floor because of their different lengths; 2) palatal plastic or cast base, which can be connected with molars or premolars to produce different anchorage effects.

Previous studies have shown that the midpalate suture does not fuse completely under sustained mechanical force, which makes skeletal expansion possible in nongrowing patients [11]. In a retrospective study, Cho et al. found that patients whose midpalate suture had been fused had an increase in midpalate suture width after MARPE treatment [12]. Mehta et al. found an increase in nasal width after MARPE treatment in patients aged 11–15 years; the effects were stable in the short- and long-term [13]. Kim reported that a pediatric patient with OSAHs and skeletal Class III malocclusion who underwent MARPE showed considerable improvements in breathing and facial morphology after 13 years of follow-up [14]. Although some studies have evaluated airway response after MARPE treatment, the exact effects on nongrowing patients remain unknown. The goal of orthodontic treatment is not only a stable occlusal relationship, but also the coordinated beauty of the maxillofacial area. Previous studies have shown that palatal expansion techniques impact the facial soft tissues and lead to an increase in alar width and midfacial changes [15, 16]. However, there are differing opinions on the precise effects of treatment on MARPE. Akan et al. found that facial height and upper lip length increased after skeletal expansion in adolescents [17], but the results reported by An et al. suggest the opposite [18]. Krijt et al. reported that after MARPE treatment, there was a significant anterior movement in the regions of the nose, left of philtrum, right of philtrum, and upper lip tubercle, while there was no significant increase in alar width [19].

Therefore, the short- and long-term effects of MARPE on the upper airway and alar among nongrowing patients remain unclear. The objective of this systematic review and meta-analysis study was to evaluate the short- and long-term changes in the upper airway and alar in nongrowing patients treated with MARPE.

## Methods

### Protocol and registration

This meta-analysis was conducted in accordance with the Preferred Reporting Items for Systematic Reviews and Meta-Analysis (PRISMA) statement guidelines. The Prospero registration number for the study protocol is CRD42023406225.

### Eligibility criteria

As shown in Table 1, the PICOS principle was used to construct the inclusion and exclusion criteria.

**Table 1:** Inclusion and exclusion criteria: PICOS framework

**Inclusion criteria**	**Exclusion criteria**
**Participants (P)**	
- Adult, Non-growing, > 15 years old - Maxillary transverse deficiency	- Children, Growing, < 15 years old - Systemic disease/craniofacial anomalies/syndrome
**Intervention (I)**	
- Non orthognatic surgery - 2 or 4 micro-implant assisted rapid maxillary expansion - MARPE, MSE, MARME	- In vitro/Laboratory/Molecular/Cellular/Animal-Surgery - Finite element study - RME, RPE, SARPE, SARME - Bone distraction - Tooth borne RME
**Comparison (C):**	
-compared vs. post treatment or MARPE vs. SARPE or RPE	
**Outcome measures (O)**	
- Cone-beam computed tomography (CBCT) - The changes in the upper airway and facial soft tissue	
**Study design (S)**	
- Randomized controlled trial (RCT) - Cohort study - Case–control study - Analytic cross-sectional study - Descriptive study - One-group pretest–posttest design	- Case report/ Case series/opinions/Letter to editor - Narrative review/summary - Systematic review/Meta-analysis

*RME* Rapid maxillary expansion, *RPE* Rapid palatal expansion, *MARME* Micro-implant assisted rapid maxillary expansion, *MARPE* Mini-screw assisted rapid palatal expansion, *MSE* Maxillary skeletal expansion, *SARME* Surgically-assisted RME, *SARPE* surgically-assisted RPE

### Search strategy

The PubMed, Embase, Scopus, Web of Science, and Cochrane Library electronic databases were searched up to 2 August, 2023 without date or language restrictions. Unpublished studies were eligible for inclusion. The reference lists of previous systematic reviews and meta-analyses were also manually searched. Search strategy was formulated and the details are shown in Appendix Table 1.

### Study selection

Duplicate documents were removed with Endnote X9. Based on the title and abstract information of the articles, the two reviewers (LCY and WKX) independently screened out the articles that met the criteria, and then reviewed the full texts of the potentially eligible articles to select those that ultimately met the inclusion criteria. Disagreements between reviewers were resolved by consensus with a third reviewer (LY).

### Data collection

Data extraction was independently performed by two reviewers (JCM/ZY) according to full text inclusion/exclusion criteria. When there were insufficient data in the articles, we contacted the authors by e-mail for additional information. Any disagreements between the two reviewers were resolved by discussion with a third reviewer (FCH). The following data were extracted from the included studies: sample size, sex, mean age, type of study, appliance type, quantity, length and diameter of the mini-implants, activation protocol, activation duration and measurement time.

Due to different follow-up times in the included studies, we pooled them into three time points: before treatment (T0), within 3 months after expansion (T1), and more than 3 months after expansion (T2). Changes in the main outcomes over time periods T1-T0, T2-T1 and T2-T0 were extracted and quantified as the mean difference and 95% confidence intervals. The main outcomes were divided into 3 parts:

- Nasal cavity changes: nasal cavity width, nasal floor width, and nasal cavity volume;
- Upper airway volume changes: nasopharyngeal volume, oropharyngeal volume, hypopharyngeal volume and total volume. oropharyngeal volume was further divided into palatopharyngeal volume and glossopharyngeal volume;
- Alar changes: alar width, alar base width.

### Risk of bias assessment individual studies

Two reviewers (ZYY/ZQW) independently assessed the quality of 24 articles. Any disagreements were resolved by discussion with the third reviewer (LY). The criteria were modified from the method reported by Jing Huang on the basis of the CONSORT statement [15]. The detailed criteria for risk of bias assessment are mainly divided into the following 6 aspects with 17 articles:

1. Study samples: age and gender distribution described (1), clinical features fully defined (1), sample size: adequate (1).
2. Study design: presence of a blank control (1), prospective (1), randomization (1).
3. Treatment details: appliances described (1), interventions fully described (1), follow-up defined (1).
4. Study measurements: measurement method: appropriate (1), assessor blinding (1), reliability testing (1).
5. Study data: no dropouts or explained (1), statistical analysis: appropriate (1), confounders analyzed (1).
6. Study results: results reported: adequate (1), reasonable conclusion (1).

Each item is scored as 1 if it meets the criteria and 0 if it does not. In total, the maximum sum was 17 points; scores of ≥ 15, scores of < 15 and ≥ 12 and scores of < 12 were considered to represent high, moderate, and low quality, respectively.

### Summary measures and synthesis of results

Summary measures and synthesis of results were independently performed by two reviewers (LCY/WKX). Stata MP 17.0 was used for data analysis. The mean difference and 95% confidence interval (CI) were used for continuous data. I^2^ statistics were used to test the statistical heterogeneity with a significance level of α = 0.05. If the I^2^ value was greater than 50%, there was high heterogeneity and a random effects model was adopted. Then further explore the reasons for high heterogeneity in the results, including analyzing study quality and conducting subgroup analysis based on different sample characteristics and treatment methods, such as patient age, gender, degree of palatal suture ossification and staging, palatal bone thickness, length of dental implants, and presence of dental anchorage etc. [20–24]. When the I^2^ value was lower than 50%, a fixed effects model was used. Based on the main outcomes, forest plots were made for visual analysis. Egger tests were used to quantitatively assess publication bias with a significance level of α = 0.05, and funnel plots were used for visual evaluation. Sensitivity analyses were performed to assess the robustness of the main outcomes [25]. T1-T0, T2-T1 and T2-T0 were used for subgroup analysis.

## Result

### Study selection

**Fig. 1:**
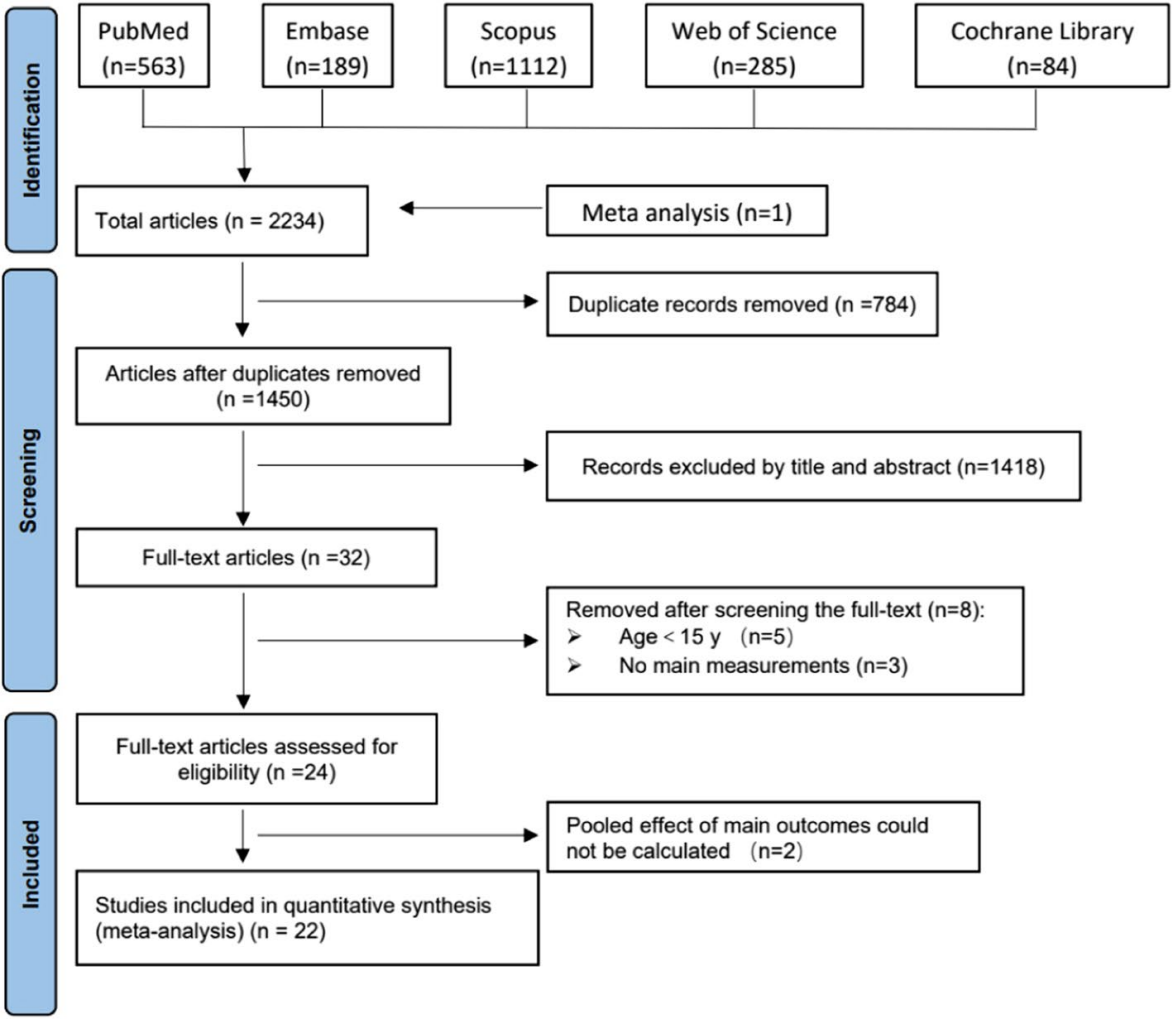
The PRISMA flow diagram

As shown in Fig. 1, the PRISMA flow diagram shows the study selection process. The initial results from the 5 databases were as follows: PubMed, 563; Embase, 189; Scopus, 1112; Web of Science, 285; and Cochrane Library, 84. A total of 2234 studies were included, and 1450 were obtained after removing 784 duplicates. After screening titles and abstracts, 1418 articles were excluded and 32 studies were included for full text analysis. One study [26] was obtained by manually searching the reference list of the study by Kapetanović et al. [27]. A total of 24 studies were included after examining the full texts, 22 of which were included for quantitative analysis.

### Study characteristics

Main characteristics of the included studies in this systematic review are shown in Table S1. Twelve studies were of the retrospective one-group pretest–posttest design [23, 28–38]. Eight studies were prospective [19–22, 39–42] and four studies were retrospectively compared with other arch expansion methods [18, 26, 43, 44]. The 24 studies included a total of 599 patients, 240 women and 320 men. Three studies did not report information on gender [35, 40, 44]. The average age of the patients was over 15 years old. For the treatment protocol, 4 min-screws in the palate are used, but the diameter, length and type of appliance are different. The activation protocol was 1 or 2 turns (0.13–0.25 mm per turn) per day or every other day. The definition of successful expansion varied slightly across the included studies, but it was commonly considered adequate when the palatal cusps of the maxillary first molars touched the buccal cusps of the mandibular first molars.

### Bias assessment

The results of the bias assessment of the 24 included studies are shown in Table S2. Three studies were of low quality due to insufficient sample sizes and no description of sex characteristics [35, 40, 44]. Only one high-quality study was conducted [22]. Most of the remaining studies were retrospective, so the quality was moderate. Six studies conducted posttreatment follow-up [19, 28, 30, 31, 33, 41]. Seven studies used assessor blinding [18, 20, 22, 23, 30, 42, 43].

### Meta-analysis

Measurement results of nasal cavity, upper airway and alar changes are shown in Table S3. Forest plots of the measurement results in T1-T0, T2-T1, and T2-T0 are shown in Figures S1, S2, S3, S4, S5, S6, S7, S8, S9, S10 and S11. The vertical line of the diagram indicates the point estimate of the means and the horizontal line shows the 95% CI. The pooled mean estimate with 95% CI of the nasal cavity, upper airway and alar changes are shown in Table S4.

Heterogeneity tests showed that the *p* values of the volume of the nasopharynx, palatopharynx, glossopharynx, oropharynx and hypopharynx were not statistically significant (*p* > 0.05), so a fixed effects model was used (I^2^<50%). For other main outcomes, a random effects model was used. Owing to the constraints imposed by the number of studies encompassed, further subgroup analyses is restricted solely to the width of the nasal cavity. Among the included studies, the divergence in key attributes resides in mini screws length and the activation protocols. As exemplified in Figures S12, S13 and S14, the outcomes of the subgroup analysis evinced statistical significance with a *P*-value <0.05, thereby intimating that the length of mini screws and the activation protocol exert an influence on MARPE treatment. It is discerned that longer mini-screws and a twice-daily activation regimen may engender more pronounced expansion.

Publication bias was assessed for indicators greater than 3 of the included studies. As shown in Figures S15, S16, S17, S18, S19, S20, S21, S22, S23, S24, S25, S26, S27, S28, S29, S30, S31 and S32 and Table S5, visual and quantitative assessments were performed using funnel plots and Egger tests respectively. The results revealed the non-significant *p* value of the parameters (*P* > 0.05), no significant publication bias was considered. The sensitivity analysis results are shown in Table S6 and Figures S33, S34, S35, S36, S37, S38, S39, S40, S41, S42, S43, S44, S45, S46, S47, S48, S49, S50, S51, S52, S53, S54 and S55. We found that the pooled mean estimates with 95% CIs of nasal cavity volume, oropharyngeal volume, hypopharyngeal volume and total volume were strongly influenced by the included studies and may have heterogeneity. The results for other parameters were more robust and were less affected by the results of the included studies.

## Discussion

### Summary of evidence

The objective of this meta-analysis was to assess short- and long-term changes in the upper airway and alar width in nongrowing patients older than 15 years of age who received MARPE. A total of 24 studies met the eligibility criteria and 22 studies were included in a quantitative analysis.

### Changes in nasal cavity width, nasal floor width and nasal cavity volume

According to the results of the present study, nasal cavity width (WMD: 2.10 mm, 95% CI: 1.73, 2.47) and nasal floor width (WMD: 2.43 mm, 95% CI: 1.87, 2.99) increased at T1-T0. Although nasal cavity width (WMD: -0.29 mm, 95% CI: -0.44, -0.13) relapsed to some extent at T2-T1, nasal cavity width (WMD: 2.81 mm, 95% CI: 1.09, 4.53) and nasal floor width (WMD: 2.37 mm, 95% CI: 1.67, 3.07) increased at T2-T0. Lim et al. also reported that nasal floor width (mean: -0.64 mm, 95% CI: -0.93, -0.35) decreased at T2-T1 [32].

Only one study reported an increase in nasal cavity volume (mean: 1.06 cm^3^, 95% CI: 0.74, 1.38) at T1-T0. Nasal cavity volume (WMD: 1.88 cm^3^, 95% CI: 1.05, 2.72) also increased at T2-T0. Inconsistent with the results of a decrease in nasal cavity width (WMD: -0.29 mm, 95% CI: -0.44, -0.13) at T2-T1, Kim et al. reported an increase in nasal cavity volume (mean: 0.65 cm^3^, 95% CI: 0.22, 1.08) over this time period [31].

MARPE is generally maintained for 3 months after treatment and corresponding soft tissue modifications after skeletal tissue changes take more time, which may be the cause of width recurrence from T2-T1. Cameron et al. reported an increase in nasal cavity width can effectively enlarge the nasal cavity volume to improve respiratory function, and it was maintained after 8 years of follow-up [45]. Arqub et al. systematically found that increased nasal cavity width after MARPE treatment can reduce respiratory resistance [46]. There was a high level of heterogeneity for width changes, partly due to different activation protocols, with three studies [30, 31, 36] activating one turn per day, two studies[37, 39] activating one turn every other day, and the remaining four studies activating two turns (0.25 mm per turn) per day. Nonetheless, it must be noted that there is currently limited research on the efficacy of MARPE treatment in nongrowing patients. Earlier investigations indicate that both rapid and slow expansion protocols yield comparable overall effects [47, 48]. Furthermore, the subgroup analysis further underscores that the length of mini screws can affect the changes in nasal cavity width after MARPE treatment. This observation aligns with the findings of Choi's study, which suggest that the longer implants may increase the amount of skeletal expansion due to their bio-cortical bone anchorage [22].

The sensitivity analysis revealed that the findings were robust and unaffected by the included studies, so the increase in nasal cavity width and nasal floor width after MARPE treatment was significant. However, the results of nasal cavity volume in T2-T0 were strongly influenced by the included studies and the results were not robust. Therefore, conclusions should be drawn with caution regarding the increase in nasal cavity volume after MARPE treatment. Higher quality RCTs are needed to investigate the impact of MARPE on nasal cavity volume.

### Changes in upper airway volume

In this study, nasopharyngeal volume (WMD: 0.75 cm^3^, 95% CI: 0.44, 1.06) increased at T2-T0. There was no significant heterogeneity, I^2^ = 32.5% (*P* > 0.05). Sensitivity analysis revealed that the results were stable, so the effect of MARPE treatment on nasopharyngeal volume was statistically significant.

Shetty et al. reported that there was no significant change in palatopharyngeal and glossopharyngeal volume immediately after expansion [35]. The results of our systematic analysis suggested that palatopharyngeal volume (WMD: 0.51 cm^3^, 95% CI: -0.25, 1.27) and glossopharyngeal volume (WMD: 0.34 cm^3^, 95% CI: -0.26, 0.94) remained unchanged at T2-T0. We anatomically defined the oropharynx as being divided into the palatopharynx and glossopharynx. Similar to the palatopharyngeal and glossopharyngeal volume changes at T2-T0, oropharyngeal volume (WMD: -0.49 cm^3^, 95% CI: -3.62, 2.65) did not change significantly at T1-T0, but it (WMD: 0.92 cm^3^, 95% CI: 0.50, 1.33) increased at T2-T0. Kim et al. reported an increase in oropharyngeal volume at T2-T1 [31]. These inconsistent results may be due to differences in the definition of anatomical boundaries, the activation protocol and the thickness of the implant through the cortex used. Kim et al. [31] used the choanae and the third cervical vertebrae as the boundary, while the other 5 studies [20, 32, 35, 36, 38] used the posterior nasal spine (PNS) and epiglottis as the boundary. Tang et al. [36] activated 1 turn(0.2 mm) per day, and other studies[31, 32, 35, 38] activated 2 turns(0.25 mm) a day. The different mandibular positions due to upright and supine positions when taking CBCT may be responsible for the contradictory results. Tang et al. [36] placed the patient in the supine position when performing CBCT, Aneris, Kim and Yi et al. [20, 31, 38] placed the patient in the upright position. There are studies reporting that the use of different appliance materials can affect the effectiveness of orthodontic treatment such as self-curing plastics or cast metals, molar anchorage [49, 50].

In addition, the sensitivity analysis showed that the oropharyngeal volume was greatly influenced by the included studies. Therefore, the results were unstable and due to the small number of included articles and varying quality, it is cautionary to draw conclusions that oropharyngeal volume was increased at T2-T0 despite the results of meta-analysis suggesting an increase. Previous meta-analyses have shown that nasal cavity volume increased and oropharyngeal volume remained unchanged after MARPE, but they included pediatric patients, so the confounding factors of growth and development cannot be ruled out [51].

Two articles described no significant changes in hypopharyngeal volume (WMD: -0.90 cm^3^; 95% CI: -1.86, 0.06) and the difference was not statistically significant (*P* > 0.05) [32, 36]. This is consistent with previous studies that showed no significant change in the volume of the inferior section of the upper airway after MARPE treatment [52]. Total volume (WMD: 1.67 cm^3^, 95% CI: 0.68, 2.66) increased at T2-T0.

Li et al. reported that MARPE can produce more transverse skeletal expansion, relieve maxillary transverse deficiency and improve upper airway ventilation [32]. Tang et al. found that respiratory resistance, speed, minimum shear force and other respiratory functions of adult patients improved after MARPE treatment, which mainly relied on their anatomical changes [36]. Studies have found that improved airflow characteristics in patients with sleep apnoea syndrome after MARPE treatment are significantly associated with improvements in polysomnography results, suggesting that MARPE is a viable treatment option [2]. However, Arqub et al. reported that there was no correlation between upper airway changes and airway ventilation in a systematic analysis [46]. Ronchi et al. found that mandibular setback surgery results a statistically significant posterior airway space reduction in the medium- and long-term follow-up. But, no direct correlation was identified with OSAS risk [53]. Due to the small number of included articles, more high-quality RCTs are needed to explore the relationship between upper airway changes and respiratory function.

### Alar width changes

Based on the results of this study, alar width (WMD: 1.47 mm; 95% CI: 0.40, 2.55) and alar base width (WMD: 1.62 mm; 95% CI: 1.11, 2.13) increased in T1-T0. The difference was statistically significant (*P* < 0.05) and the changes in both were consistent with the corresponding skeletal nasal cavity width changes. However, heterogeneity analyses revealed that I^2^ > 80%, and sensitivity analyses showed robust results, possibly due to inconsistent measurements in the included studies. Jesus et al. [44] and Shetty et al. [35] used CBCT three-dimensional (3D) reconstruction, Krijt et al. and Lee et al. used 3D stereophotogrammetry [19, 39]. An et al. used 2D frontal photos to find that the alar width also increased [18]. 3D measurement of facial soft tissues is a new direction that can avoid the superimposition and image distortion observed with the 2D radiography technique [28]. Staller et al. found that there are some differences between 2D photos and 3D measurements. However, this error is clinically acceptable [54]. Lavorgna also reported that there were no significant differences emerged in the measurements made with 3D stereophotogrammetry and photogrammetry [55].

From the perspective of clinical methodology, the activation protocol was as follows: Jesus et al. (0.5 mm/d) > Krijt et al. (0.25 mm/d) > Lee et al. (0.2 mm/d), which may be one of the reasons for the high heterogeneity of alar width. Therefore, orthodontists should pay attention to the effect of activation strategies on alar changes. This may affect the aesthetics of the nose. Brito et al. believed that the nasal framework basically determines nasal morphology, and nasal morphology changes when the skeletal nasal cavity changes [56]. In patients with a depression in the middle of the face, the anterior movement of the maxilla can improve the facial shape, but the increase in alar width and alar base width may affect the aesthetics of the nose, resulting in a collapsed nose and a humped nose [15]. Abedini et al. used 3DMD to analyse facial soft tissue and reported significant forwards sagittal and lateral asymmetry changes in the paranasal, upper lip, both cheeks and with greater changes in the cheek area. Those changes remained stable after 1 year [28]. An et al. found that the maxilla A point was significantly forwarded by 1.3 mm on average, and the length of the nose and upper lip increased, but this increase was not statistically significant [18]. Shetty et al. also found an increase in the H-angle after MARPE treatment, which may be related to the forwards shift of the A point [35]. Almaqrami et al. reported that the amount of maxillary forwards movement was small (0.88°), which might not be clinically significant and the mandible rotated downwards and backwards. However, Nguyen et al. found more significant lateral changes in paranasal and cheek areas [34].

The goal of orthodontic treatment is not only the alignment of teeth and a stable occlusal relationship, but the beauty and coordination of the face is also one of the goals pursued by orthodontists and patients. The coordination of the nose, lips and chin is an important parameter index to evaluate the results of orthodontic treatment. The effect of MARPE on the soft tissues has rarely been studied, which could be due to the assumption that it is expected to be minute, or overshadowed by other growth changes and therefore hard to evaluate. However, with the introduction of MARPE, more nongrowing patients can be expanded skeletally, which calls for a more in-depth study on the actual effects of expansion on soft tissues.

### Strengths and limitations

A large number of studies have focused on skeletal and dental changes after MARPE treatment, with limited research on the upper airway and facial soft tissue. Patients treated with MARPE are usually in the advanced stages of growth, therefore the use of traditional expanders such as Haas Hyrax is not indicated. Compared with the meta-analysis of Li et al., we did not include studies with case groups under 15 years of age and excluded growth as a confounding factor [51]. This meta-analysis was conducted in accordance with the Preferred Reporting Items for Systematic Reviews and Meta-Analyses (PRISMA) statement guidelines. We strictly defined the inclusion and exclusion criteria in accordance with the PICOS principles. We used follow-up time as a subgroup and performed detailed heterogeneity tests, publication bias, and sensitivity analyses.

The quality of the literature included in this review was mostly moderate and most of them were retrospective one-group pretest–posttest study designs with a small sample size (< 25). It is difficult to conduct high-quality RCTs due to clinical ethical issues. Heterogeneity was high, but the number of studies did not allow for relevant subgroup analyses and sensitivity tests. More studies need to be included for analysis due to the potential confounding factors of race, sex, appliance type, diameter and length of implant nails, etc. The anatomical boundaries and measurement methods of the included studies were also not completely consistent. Most of the follow-up included in this study was within one year, and it was difficult to assess longer-term changes after MARPE treatment, so longer follow-up studies are encouraged.

## Conclusions

The nasal width and nasal base width of skeletal and soft tissues increased. The volumes of the nasal cavity, nasopharynx, and oropharynx increased, but the volumes of the palatopharynx, glossopharynx, and hypopharynx remained unchanged. However, due to the number of included studies and high heterogeneity, these conclusions should be made with caution and require more higher-quality RCTs to investigate the relationship between expansion strategies and treatment outcomes.

### Supplementary Information

**Additional file 1: Appendix table 1. **The details of search strategy.

**Additional file 2: Table S1.** Main characteristics of the included studies in this systematic review.

**Additional file 3: Table S2.** The results of the bias assessment of the 24 included studies.

**Additional file 4: Table S3.** Measurement results of the nasal cavity, upper airway and alar changes.

**Additional file 5: Table S4.** The pooled mean estimate with 95% CI of the nasal cavity, upper airway and alar changes.

**Additional file 6: Table S5. **Egger tests of the nasal cavity, upper airway and alar changes.

**Additional file 7: Table S6. **The sensitivity analysis results of the nasal cavity, upper airway and alar changes.

**Additional file 8: Figure S1-S11. **Forest plots of the measurement results in T1-T0, T2-T1, and T2-T0.** Figure S12-S14. **Forest plots of nasal cavity width based on activation protocol and length of mini screws in T1-T0 and T2-T0.

**Additional file 9: Figure S15-S32.** Funnel plots of the nasal cavity, upper airway and alar changes.

**Additional file 10: Figure S33-S55. **The sensitivity analysis results of the nasal cavity, upper airway and alar changes.

## Data Availability

The datasets supporting the conclusions are included in the article and its additional files.

## Abbreviations

MARPE: Mini-Implant Assisted Rapid Palatal Expansion
MTD: Maxillary transverse deficiency
OSAHS: Obstructive sleep apnea-hypopnea syndrome
RPE: Rapid palatal expansion
SARPE: Surgically assisted RPE
RCTs: Randomized controlled trials
MD: Mean difference
WMD: Weighted mean difference
CI: Confidence intervals
PRISMA: Preferred Reporting Items for Systematic Reviews and Meta-Analysis
RPE: Rapid palatal expansion
MARME: Micro-implant assisted rapid maxillary expansion
MSE: Maxillary skeletal expansion
SARME: Surgically-assisted RME
