# Supplementary material for: The short- and long-term changes of upper airway and alar in nongrowing patients treated with Mini-Implant Assisted Rapid Palatal Expansion (MARPE): a systematic review and meta-analysis
Source: BMC Oral Health. 2023 Oct 29;23:820. doi: 10.1186/s12903-023-03344-w (PMC10613376; doi:10.1186/s12903-023-03344-w)
Supplement: Supplementary file 8 — Additional file 8: Figure S1-S11. Forest plots of the measurement results in T1-T0, T2-T1, and T2-T0. Figure S12-S14. Forest plots of nasal cavity width based on activation protocol and length of mini screws in T1-T0 and T2-T0. [file 12903_2023_3344_MOESM8_ESM.docx]

**Figure S1-S11**. Forest plots of the measurement results in T1-T0, T2-T1, and T2-T0.

**Figure S12-S14.** Forest plots of nasal cavity width based on activation protocol and length of mini screws in T1-T0 and T2-T0.

**Figure S1．**Forest plot for the changes of nasal cavity width

**Figure S2**. Forest plot for the changes of nasal floor width.

**Figure S3.** Forest plot for the changes of nasal cavity volume.

**Figure S4.** Forest plot for the changes of nasopharyngeal volume.

**Figure S5.** Forest plot for the changes of palatopharyngeal volume

**Figure S6.** Forest plot for the changes of glossopharyngeal volume.

**Figure S7.** Forest plot for the changes of oropharyngeal volume.

**Figure S8.** Forest plot for the changes of hypopharyngeal volume.

**Figure S9.** Forest plot for the changes of total volume.

**Figure S10.** Forest plot for the changes of alar width.

**Figure S11.** Forest plot for the changes of alar base width.

**Figure S12.** Nasal cavity width based on activation protocol.

**Figure S13.** Nasal cavity width based on length of mini screws in T1-T0.

**Figure S14**. Nasal cavity width based on length of mini screws in T2-T0.
