# Supplementary material for: The short- and long-term changes of upper airway and alar in nongrowing patients treated with Mini-Implant Assisted Rapid Palatal Expansion (MARPE): a systematic review and meta-analysis
Source: BMC Oral Health. 2023 Oct 29;23:820. doi: 10.1186/s12903-023-03344-w (PMC10613376; doi:10.1186/s12903-023-03344-w)
Supplement: Supplementary file 9 — Additional file 9: Figure S15-S32. Funnel plots of the nasal cavity, upper airway and alar changes. [file 12903_2023_3344_MOESM9_ESM.docx]

**Figure S15-S32.** Funnel plots of the nasal cavity, upper airway and alar changes

**Figure S15.** Funnel plots of nasal cavity width.

**Figure S16.** Funnel plots of nasal cavity width in T1-T0.

**Figure S17.** Funnel plots of nasal cavity width in T2-T0.

**Figure S18.** Funnel plots of nasal floor width

**Figure S19.** Funnel plots of nasal floor width in T1-T0

**Figure S20.** Funnel plots of nasal floor width in T2-T0

**Figure S21.** Funnel plots of nasal cavity volume.

**Figure S22.** Funnel plots of nasopharyngeal volume.

**Figure S23.** Funnel plots of palatopharyngeal volume.

**Figure S24.** Funnel plots of palatopharyngeal volume T2-T0.

**Figure S25.** Funnel plots of glossopharyngeal volume.

**Figure S26.** Funnel plots of glossopharyngeal volume T2-T0.

**Figure S27.** Funnel plots of oropharyngeal volume.

**Figure S28.** Funnel plots of oropharyngeal volume in T2-T0.

**Figure S29.** Funnel plots of total volume.

**Figure S30.** Funnel plots of total volume in T2-T0.

**Figure S31.** Funnel plots of alar base width.

**Figure S32.** Funnel plots of alar base width in T1-T0.
