# Supplementary material for: The short- and long-term changes of upper airway and alar in nongrowing patients treated with Mini-Implant Assisted Rapid Palatal Expansion (MARPE): a systematic review and meta-analysis
Source: BMC Oral Health. 2023 Oct 29;23:820. doi: 10.1186/s12903-023-03344-w (PMC10613376; doi:10.1186/s12903-023-03344-w)
Supplement: Supplementary file 10 — Additional file 10: Figure S33-S55. The sensitivity analysis results of the nasal cavity, upper airway and alar changes. [file 12903_2023_3344_MOESM10_ESM.docx]

**Figure S33-S55.** The sensitivity analysis results of the nasal cavity, upper airway and alar changes

**Figure S33.** The sensitivity analysis results of the nasal cavity width.

**Figure S****34.** The sensitivity analysis results of the nasal cavity width in T1-T0.

**Figure S35.** The sensitivity analysis results of the nasal cavity width in T2-T1.

**Figure S36.** The sensitivity analysis results of the nasal cavity width in T2-T0.

**Figure S37.** The sensitivity analysis results of the nasal floor width.

**Figure S38.** The sensitivity analysis results of the nasal floor width in T1-T0.

**Figure S39.** The sensitivity analysis results of the nasal floor width in T2-T0.

**Figure S40.** The sensitivity analysis results of the nasal cavity volume.

**Figure S41.** The sensitivity analysis results of the nasal cavity volume in T2-T0.

**Figure S42.** The sensitivity analysis results of the nasopharyngeal volume.

**Figure S43.** The sensitivity analysis results of the palatopharyngeal volume.

**Figure S44.** The sensitivity analysis results of the palatopharyngeal volume in T2-T0.

**Figure S45.** The sensitivity analysis results of the glossopharyngeal volume.

**Figure S46.** The sensitivity analysis results of the glossopharyngeal volume in T2-T0.

**Figure S47.** The sensitivity analysis results of the oropharyngeal volume.

**Figure S48.** The sensitivity analysis results of the oropharyngeal volume in T1-T0.

**Figure S49.** The sensitivity analysis results of the oropharyngeal volume in T2-T0.

**Figure S50.** The sensitivity analysis results of the hypopharyngeal volume.

**Figure S51.** The sensitivity analysis results of the total volume.

**Figure S52.** The sensitivity analysis results of the total volume in T2-T0.

**Figure S53.** The sensitivity analysis results of alar width in T1-T0.

**Figure S54.** The sensitivity analysis results of alar base width.

**Figure S55.** The sensitivity analysis results of alar base width in T1-T0.
